# Bias towards publishing positive results in orthopedic and general surgery: a patient safety issue?

**DOI:** 10.1186/1754-9493-1-4

**Published:** 2007-11-27

**Authors:** Erik A Hasenboehler, Imran K Choudhry, Justin T Newman, Wade R Smith, Bruce H Ziran, Philip F Stahel

**Affiliations:** 1 Department of Orthopedic Surgery, Denver Health Medical Center, University of Colorado School of Medicine, 777 Bannock Street, Denver, CO 80204, USA; 2 Department of Orthopedic Trauma, Northeast Ohio Universities College of Medicine, St. Elizabeth Health Center, 1044 Belmont Ave, Youngstown, OH 44501, USA

## Abstract

**Background:**

Research articles reporting positive findings in the fields of orthopedic and general surgery appear to be represented at a considerably higher prevalence in the peer-reviewed literature, compared to published studies on negative or neutral data. This "publication bias" may alter the balance of the available evidence-based literature and may affect patient safety in surgery by depriving important information from unpublished negative studies.

**Methods:**

A comprehensive review of all published articles in a defined 7-year period was performed in 12 representative journals in the fields of orthopedic and general surgery. Every article published in all volumes of these journals between January 2000 and December 2006 was reviewed and rated by three investigators. Rating of articles was performed according to a uniform, standardized algorithm. All original articles were stratified into "positive", "negative" or "neutral", depending on the reported results. All non-original papers were excluded from analysis.

**Results:**

A total of 30,197 publications were reviewed over a 7-year time-period. After excluding all non-original articles, a total of 16,397 original papers were included in the final analysis. Of these, 12,251 (74%) articles were found to report positive findings, 2,709 (17%) reported negative results, and 1,437 (9%) were neutral. A similar publication pattern was found among all years and all journals analyzed. Altogether, 91% of all original papers reported significant data (positive or negative), whereas only 9% were neutral studies that did not report any significant findings.

**Conclusion:**

There is a disproportionately high number of articles reporting positive results published in the surgical literature. A bias towards publishing positive data will systematically overestimate the clinical relevance of treatment effects by disregarding important information derived from unpublished negative studies. This "publication bias" remains an area of concern and may affect the quality of care of patients undergoing surgical procedures.

## Introduction

Peer-reviewed biomedical journals are more likely to publish original papers reporting positive results than studies with negative data [1-4]. This "publication bias," also termed "positive-outcome-bias," has been recognized and described in the internal medicine literature [5-8]. In addition, commercial funding of clinical studies has been described as an independent variable associated with the frequency of publication of positive articles [9-11]. Evidence-based decision making processes on therapeutic modalities rely on the availability of unbiased, balanced, and objective data from published studies, independent of the reported outcome [12]. Particularly, high-quality systematic meta-analyses are corrupted by the "positive-outcome-bias" of individual studies, rendering clinical recommendations flawed towards a positive effect of specific treatment strategies [13,14]. In the era of evidence-based medicine, this prevalent, often unrecognized, publication bias poses a severe challenge to patient safety by promoting unjustified therapeutic concepts [13,15-17]. This notion is particularly true in surgical disciplines, since specific surgical techniques are frequently adapted or abandoned depending on the current status of evidence-based recommendations in the scientific literature [18-20].

Despite its potentially devastating clinical impact, the prevalence of a "publication bias" remains largely unexplored in the field of surgery and surgical subspecialties [9,21-23]. The present study was designed to analyze a potential "positive outcome bias" in 12 representative orthopedic and general surgery journals over a time-period of 7 years. We hypothesized that studies reporting positive outcomes are represented in the surgical literature at considerably higher prevalence than those which report negative or neutral findings.

## Methods

A comprehensive review of 12 selected journals from the disciplines of orthopedic and general surgery was systematically performed in all published volumes between January 2000 and December 2006. The representative journals within these disciplines were selected by the senior author. Selection was based on the appraisal that these journals represent relevant sources of clinical knowledge in general and orthopedic surgery, with a wide distribution of official journal rankings and impact factors (Table 1). All co-authors concurred with the selection of these journals at the time when the study design was established.

**Table 1:** Overview on the 12 selected peer-reviewed journals analyzed in this study. Journal ranking^1 ^and journal impact factors^2 ^are derived from the *Institute for Scientific Information *(ISI) database from 2006. Journal ranking was determined by the specific subject categories of Surgery* and Orthopedics^§^. The numbers of articles analyzed in this study are shown as the overall publications^3 ^(including non-original articles) and as original articles exclusively^4^. Articles were assessed in all individual journals' volumes from 01/2000 to 12/2006.

**Journal title**	**Journal ranking^1^**	**Impact factor^2^**	**Total number of articles analyzed^3^**	**Number of original papers analyzed^4^**
***Annals of Surgery***	1*	7.678	1,835	1,277
***Journal of Bone and Joint Surgery (American)***	4^§^	2.444	3,012	1,501
***British Journal of Surgery***	5*	4.092	2,607	1,258
***Spine***	5^§^	2.351	4,357	2,860
***Journal of Bone and Joint Surgery (British)***	11^§^	1.790	2,364	1,415
***Journal of Orthopaedic Trauma***	13^§^	1.670	1,270	494
***Archives of Surgery***	14*	3.058	2,254	1,026
***Surgery***	15*	2.977	2,714	1,404
***Journal of the American College of Surgeons***	16*	2.813	2,437	866
***Journal of Trauma***	38*	2.035	3,316	1,979
***World Journal of Surgery***	45*	1.765	2,144	1,326
***Injury***	76*	1.067	1,887	991
**All journals**			**30,197**	**16,397**

**Figure 1:**
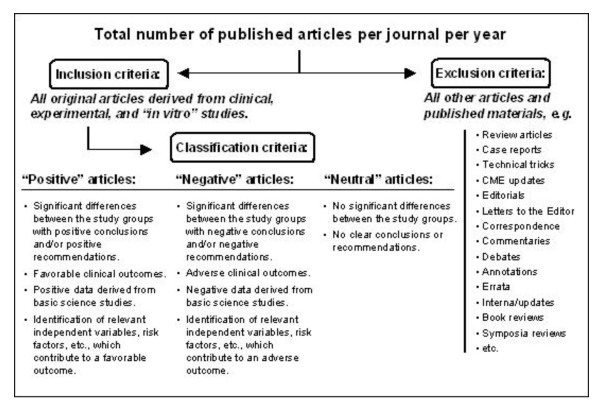
Algorithm for screening and stratification of published articles. All non-original articles were excluded from the final analysis. Original papers were classified as "positive", "negative", or "neutral" based on the outlined criteria.

The published volumes of these 12 journals from 01/2000 through 12/2006 were screened online at the individual journals' homepage. Screening and stratification of articles was performed by three investigators (EAH, IC, and JTN) according to a defined algorithm (Figure 1). Based on these criteria, each published item was counted individually and evaluated with regard to the inclusion and exclusion criteria. All non-original articles were excluded from final analysis.

Of the included original papers, the abstract was analyzed first, and categorized into either "positive," "negative" or "neutral," based on the reported results. If the abstract was unable to clearly divulge the study results, the full paper was downloaded and the manuscript's entire text was analyzed to elicit the appropriate category. Ambiguous articles were re-assessed by the senior author (PFS) who made a final decision on classification. Results were compiled for each journal and each year individually. From these numbers, the percentage of positive, negative and neutral articles was calculated as:

Number of selected original articles (positive/negative/neutral)Number of all original articles×100
 MathType@MTEF@5@5@+=feaafiart1ev1aaatCvAUfKttLearuWrP9MDH5MBPbIqV92AaeXatLxBI9gBaebbnrfifHhDYfgasaacPC6xNi=xI8qiVKYPFjYdHaVhbbf9v8qqaqFr0xc9vqFj0dXdbba91qpepeI8k8fiI+fsY=rqGqVepae9pg0db9vqaiVgFr0xfr=xfr=xc9adbaqaaeGacaGaaiaabeqaaeqabiWaaaGcbaqcfa4aaSaaaeaacqqGobGtcqqG1bqDcqqGTbqBcqqGIbGycqqGLbqzcqqGYbGCcqqGGaaicqqGVbWBcqqGMbGzcqqGGaaicqqGZbWCcqqGLbqzcqqGSbaBcqqGLbqzcqqGJbWycqqG0baDcqqGLbqzcqqGKbazcqqGGaaicqqGVbWBcqqGYbGCcqqGPbqAcqqGNbWzcqqGPbqAcqqGUbGBcqqGHbqycqqGSbaBcqqGGaaicqqGHbqycqqGYbGCcqqG0baDcqqGPbqAcqqGJbWycqqGSbaBcqqGLbqzcqqGZbWCcqqGGaaicqGGOaakcqqGWbaCcqqGVbWBcqqGZbWCcqqGPbqAcqqG0baDcqqGPbqAcqqG2bGDcqqGLbqzcqqGVaWlcqqGUbGBcqqGLbqzcqqGNbWzcqqGHbqycqqG0baDcqqGPbqAcqqG2bGDcqqGLbqzcqqGVaWlcqqGUbGBcqqGLbqzcqqG1bqDcqqG0baDcqqGYbGCcqqGHbqycqqGSbaBcqGGPaqkaeaacqqGobGtcqqG1bqDcqqGTbqBcqqGIbGycqqGLbqzcqqGYbGCcqqGGaaicqqGVbWBcqqGMbGzcqqGGaaicqqGHbqycqqGSbaBcqqGSbaBcqqGGaaicqqGVbWBcqqGYbGCcqqGPbqAcqqGNbWzcqqGPbqAcqqGUbGBcqqGHbqycqqGSbaBcqqGGaaicqqGHbqycqqGYbGCcqqG0baDcqqGPbqAcqqGJbWycqqGSbaBcqqGLbqzcqqGZbWCaaGccqGHxdaTcqaIXaqmcqaIWaamcqaIWaamaaa@AB93@

## Results

**Figure 2:**
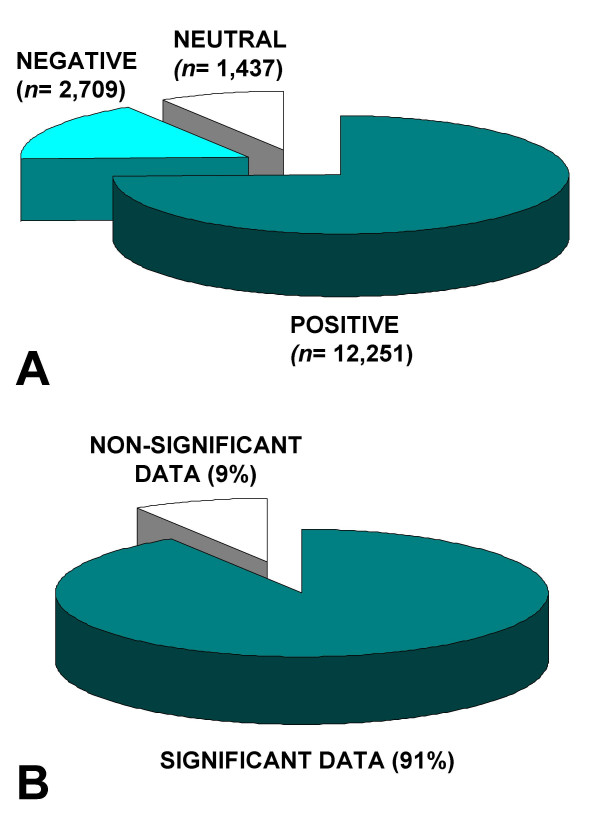
Classification of 16,397 original articles published in 12 representative peer-reviewed orthopedic and general surgery journals. **Panel A**: Percentage of publications with "positive" (*n *= 12,251; 74%), "negative" (*n *= 2,709; 17%), and "neutral" data (*n *= 1,437; 9%). **Panel B:** Percentage of publications reporting "significant" (*n *= 14,960; 91%) versus "non-significant" results (*n *= 1,437; 9%).

A total of 30,197 manuscripts published in 12 journals between January 2000 and December 2006 were reviewed. Based on the screening algorithm, 13,800 articles were classified as non-original papers and thus excluded from further analysis. The remaining 16,397 original articles were classified according the definitions outlined in the methods section (Figure 1) into "positive" studies (*n *= 12,251; 74%), "negative" studies (*n *= 2,709; 17%), and "neutral" studies (*n *= 1,437; 9%; Figure 2A). A total of 14,960 papers (91%) reported significant findings (positive and negative combined), whereas only 9% of all studies were based on data without significant differences between the groups (Figure 2B).

**Figure 3:**
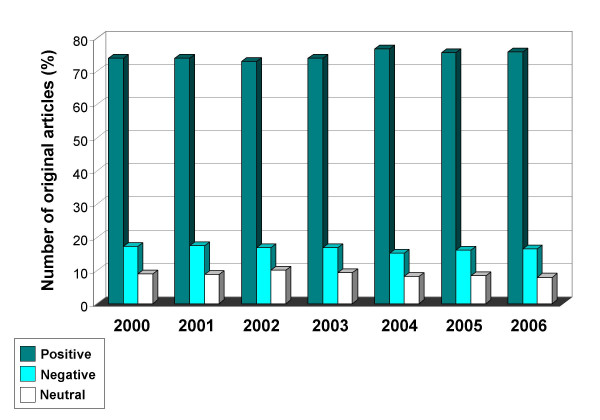
Classification of 16,397 original articles published in 12 representative peer-reviewed orthopedic and general surgery journals, stratified by publication year.

**Figure 4:**
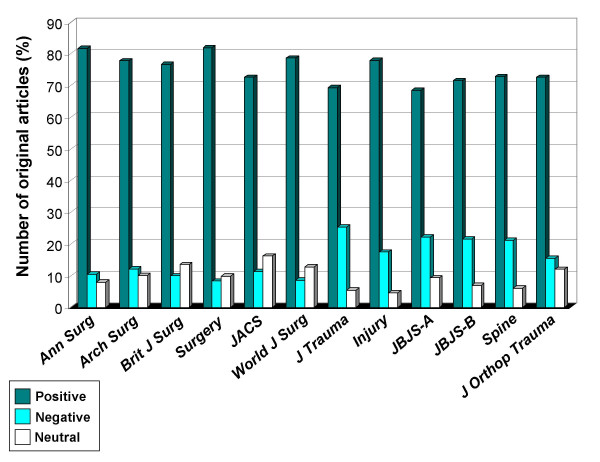
Subgroup analysis of 16,397 original articles published between 01/2000 to 12/2006, stratified by individual journal. *JACS*, Journal of the American College of Surgeons; *JBJS*, Journal of Bone and Joint Surgery (-*A*: American volume, -*B*: British volume).

There was no difference in this publication pattern over the years, from 2000 to 2006 (Figure 3). The subgroup analysis by individual journal revealed a similar publication pattern among all 12 journals, with a trend toward a higher prevalence of negative studies reported in trauma and orthopedic journals, compared to general surgery journals (Figure 4).

## Discussion

This study demonstrates for the first time, to our knowledge, a high prevalence of "positive outcome-bias" in the preeminent orthopedic and surgical literature. The disproportion of published positive studies, compared to negative or neutral papers, remained relatively constant over the seven years (2000–2006) and among the twelve journals reviewed in this study. Even more striking was the discrepancy between published original papers with significant findings (positive or negative) compared to purely "neutral" studies which did not report any significant findings (91% vs. 9%).

Our results are consistent with prior investigations which revealed a high prevalence of a "positive-outcome" publication bias in medical journals [7,15,16]. Other groups have examined the likelihood of a subsequent publication of positive versus negative studies from submitted manuscripts, unpublished manuscripts, or manuscripts derived from abstracts presented at scientific meetings [6,8,23-25]. Easterbrook et al. retrospectively reviewed 487 projects submitted for publication and found a high odds-ratio in favor of publishing articles with a statistically significant outcome compared to those manuscripts which reported no difference between the study groups [8]. Dickersin and Min performed an analysis of 198 NIH-funded trials [26]. They reported that those trials with "significant" results were more likely to be published than studies with "non-significant" data, by an adjusted odds-ratio of 12.30 [26].

In the orthopedic literature, Harris and coworkers reviewed all abstracts presented at the annual meeting American Academy of Orthopaedic Surgeons (AAOS) meeting in 1999 [25]. They found that articles with a positive outcome and significant results were more likely to be published within the following years [25]. Callaham and colleagues studied the publication pattern of abstracts presented at a 1991 major annual US research meeting in the field of emergency medicine [24]. The authors found that articles with a positive outcome were more likely to be published in peer-reviewed journals than negative studies [24].

Study funding patterns have furthermore been identified as independent factors that influence the publication bias. In particular, the presence of corporate/industrial funding was shown to be associated with a higher prevalence of published studies with positive findings. In the orthopedic literature, Leopold et al. found a significant association between commercially funded studies being more likely to have positive outcomes in publications, compared to unfunded studies [22]. This industrial funding driven bias has been confirmed in other publications [5,8,10]. Lynch et al. recently challenged this hypothesis by examining articles submitted to the American edition of the *Journal of Bone and Joint Surgery *and concluded that publications from commercially funded studies were not more likely to present positive data than non-funded studies [21]. However, this study was limited in design in that analysis was restricted to articles related to hip and knee arthroplasty during a seventeen-month period [21].

Finally, there appears to be a significant bias against negative studies in newspaper reports of medical research [17]. Thus, as for the scientist and clinician, the lay reader is also exposed to filtered information regarding the outcome of clinical studies.

The present study has a number of strengths and weaknesses. To our knowledge, it is the largest analysis of its kind in the medical literature. The 12 selected journals represent relevant sources of clinical knowledge, and are widely used as evidence-based decision-making tools in the fields of general, trauma, and orthopedic surgery. The algorithm used for stratifying articles is widely inclusive for all published original articles in the screened journals. The results were cross-checked and revisited in regular intervals by three different investigators and approved by the senior author. This allowed for a reliable, objective, and reproducible method of scoring.

Some drawbacks and limitations of this study must be addressed. Despite the uniform algorithm used for screening and classification of articles, there remains some degree of inherent inter-observer variability in the assessment of publications. The algorithm was developed by the authors for the present study and has not been externally validated by other groups. Furthermore, study analysis was limited to the last seven years in a limited number of journals in orthopedics and general surgery. Our results are neither representative for other surgical subspecialties and other fields of medicine, nor for papers published prior to the year 2,000.

A further drawback of this study is that we did not stratify between *in vitro *and experimental studies vs. clinical trials. A bias in the latter group is certainly associated with a higher risk for patients, since clinical treatment trials constitute the main basis for decision making in a clinical setting.

Another limitation is that published articles were assessed exclusively, without accounting for submitted and unpublished manuscripts. Thus, our study design does not determine whether the positive publication bias occurred at the level of manuscript submission or at the editorial decision-making level. The former notion would imply a bias towards a preferential submission of manuscripts with positive outcomes and statistically significant results; the latter would suggest a bias towards preferential publication of positive articles by peer-reviewers and editors. In this regard, Olson and colleagues revealed that the "positive-outcome-bias" does not occur at the editorial decision making stage, based on assessment of manuscripts submitted to the *Journal of the American Medical Association (JAMA) *between 1996 and 1999 [27].

Based on the large and encompassing analysis presented here, it is apparent that research reporting negative outcomes and/or statistically insignificant results is underrepresented in the surgical literature. The clinical implications of this trend are of concern due to the potential impact on patient care. The "positive-publication-bias" may alter the balance of the available evidence-based literature and may negatively affect recommendations and guidelines derived from systematic meta-analyses [13,20]. Recently, a new open-access online journal was launched which is devoted exclusively to publishing negative results in biomedicine [28]. In our opinion, it is imperative to promote the submission and publication of studies with negative outcomes and insignificant results, in order to ensure a balanced availability of evidence-based data for clinical decision-making regarding the best treatment modality for our patients.

## Competing interests

PFS is a reviewer for *Injury *and *J Trauma*. WRS and BHZ are reviewers for *JBJS-A, J Orthop Trauma, Injury*, and *J Trauma*. The authors declare no other competing interests with regard to this manuscript.

## Authors' contributions

WRS and PFS designed the study. EAH, IKC, JTN, and PFS wrote the manuscript. EAH, IKC, and JTN performed the screening and classification of published articles. PFS cross-checked the classifications. WRS and BHZ helped with final analysis of the data and editing of the manuscript. All authors read and approved the final version of the manuscript.
